# Probe Standoff Optimization Method for Phased Array Ultrasonic TFM Imaging of Curved Parts

**DOI:** 10.3390/s21196665

**Published:** 2021-10-07

**Authors:** Jorge Franklin Mansur Rodrigues Filho, Pierre Bélanger

**Affiliations:** Department of Mechanical Engineering, École de Technologie Supérieure, Montreal, QC H3C 1K3, Canada; pierre.belanger@etsmtl.ca

**Keywords:** TFM, PSF, phased array, ultrasound, standoff, curved surfaces

## Abstract

The reliability of the ultrasonic phased array total focusing method (TFM) imaging of parts with curved geometries depends on many factors, one being the probe standoff. Strong artifacts and resolution loss are introduced by some surface profile and standoff combinations, making it impossible to identify defects. This paper, therefore, introduces a probe standoff optimization method (PSOM) to mitigate such effects. Based on a point spread function analysis, the PSOM algorithm finds the standoff with the lowest main lobe width and side lobe level values. Validation experiments were conducted and the TFM imaging performance compared with the PSOM predictions. The experiments consisted of the inspection of concave and convex parts with amplitudes of 0, 5 and 15 λ_Al_, at 12 standoffs varying from 20 to 130 mm. Three internal side-drilled holes at different depths were used as targets. To investigate how the optimal probe standoff improves the TFM, two metrics were used: the signal-to-artifact ratio (SAR) and the array performance indicator (API). The PSF characteristics predicted by the PSOM agreed with the quality of TFM images. A considerable TFM improvement was demonstrated at the optimal standoff calculated by the PSOM. The API of a convex specimen’s TFM was minimized, and the SAR gained up to 13 dB, while the image of a concave specimen gained up to 33 dB in SAR.

## 1. Introduction

Interest in the ultrasonic inspection of components with complex shapes is rising rapidly. Research in phased array ultrasonic testing have shown that defects can be identified even under curved surfaces [1,2,3]. This possibility explains the increased interest in the technique among different industries, including aerospace and automotive. Since their forgings, castings, composites, and more recently, their 3D printed metals [4], will benefit greatly from a nondestructive inspection of their final and complex shape. An example of the traditional handling can be seen in the case of some aerospace forgings, where a sonic machining with a flat surface is required just for the ultrasonic inspection [5], which increases the production cost. The complexity of predicting the ultrasound path and coverage is among the difficulties that have traditionally discouraged the ultrasonic inspection of forged parts with curved and variable profiles.

However, this complexity is mitigated with the phased arrays, since they allow the control the beam profile and direction, along with a broad selection of post-processing techniques [6]. Additionally, to handle the non-flat surface profiles, studies have successfully demonstrated the possibility of using shaping sensing fibers [7], flexible array transducers [2,8,9], and even the use of ice as a coupling medium [10]. Still, water immersion along with adaptive methods has been the preferred approach of the scientific community in recent years. This approach enables an efficient imaging of the part and removes the need for wedges customized to the surface shape [11,12]. Additionally, the adaptive ultrasonic phased array inspection methods require no prior knowledge of the surface profile of the specimen under inspection [13,14]. On this approach, ultrasound transmission and imaging strategies had their capabilities demonstrated in the literature: the use of plane waves imaging methods [15,16], the real-time Dynamic Depth Full Focusing [17], the use of Virtual Source Apertures [18] and the Total Focusing Method (TFM) [3]. All which can be obtained through the post-processing of the Full Matrix Capture (FMC) [19]. Moreover, the TFM is of special interest due to its status of gold standard in terms of image quality [20]. Nevertheless, the reliability of such inspections still demands further investigation. Strong artifacts were observed in the images of some cases, probably caused by the influence of the surface profile [5,12,21,22]. Even when using the surface-adapted total focusing method (TFM), limitations were observed when imaging under sharp curvatures [21].

In a rather complex scenario, it is not only the part’s surface profile that will affect the outcome of the ultrasonic inspection, but also, a combination of all the parameters involved, such as the probe and material properties [23]. Hence, it is fundamental that further works investigate how the aforementioned limitations and image artifacts arise from these combinations of parameters (e.g., curved profiles, probe aperture, standoff, etc.). Additionally, more importantly, investigations should be performed to determine how these parameters can be designed to avoid errors in the inspection.

In this context, this paper demonstrates how the performance of the phased array immersion inspection can be optimized based on the probe standoff, in combination with the part surface profile and flaw depth. Hence, a Probe Standoff Optimization Method (PSOM) is presented, which uses the point spread function (PSF) to calculate the best probe standoff prior to inspection. Throughout the paper, the total focusing method implemented through a dual-layer media has its resulting image quality verified and compared with the PSOM predictions. The aim is to verify if the PSOM predictions agree with the results of the experimental phased array imaging of parts with concave and convex top surfaces, as well as to confirm how the optimization process improves the imaging performance.

## 2. Materials and Methods

### 2.1. Total Focusing Method Imaging

**Figure 1:**
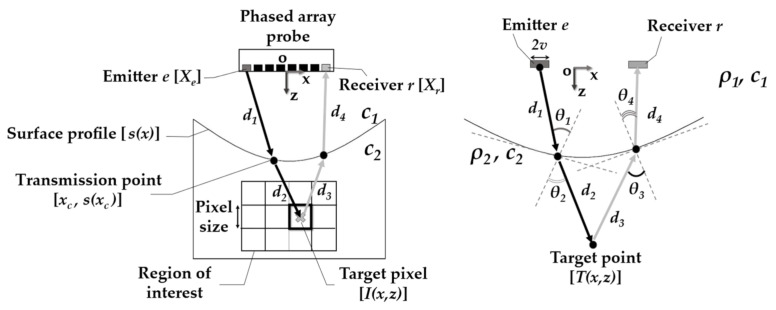
Schematic of the total focusing method applied to an immersed part with *s*(*x*) top surface profile. The waves travel from the emitter *e* to a pixel and then to the receiver *r* through the paths which respect the Fermat’s principle. The same applies for a point reflector *T*(*x*,*z*), where paths *d* have different refraction angles for each combination of *e* and *r*.

The total focusing method (TFM) is a delay-and-sum imaging algorithm that uses the full matrix capture (FMC) data acquisition scheme. Delay functions allow us to synthetically focus on every point of a region of interest inside the inspected part [19,24]. Figure 1 illustrates how the method is applied to the inspection of an immersed part having a surface profile described by *s*(*x*).

In this case, for probe elements *e* (emitter) and *r* (receiver), the target pixel *I*(*x*,*z*) in the region of interest has an amplitude given by the A-scan *S*(*e*,*r*,*t*) of the FMC data at the time advance *t = t_e_ + t_r_*. Here, *t_e_* is equal to the ultrasonic wave time-of-flight from *e* to *I*(*x*,*z*) and *t_r_* is the time-of-flight from *I*(*x*,*z*) back to *r*. By summing all the combinations of elements acting as emitter–receiver *e* and *r*, the pixel amplitude can be written as:

(1) $$I\left(x,z\right)=\left|\underset{e}{\sum }\underset{r}{\sum }S(e,r,t_{e}+t_{r})\right|$$

As there are two different wave propagation velocities *c_1_* and *c_2_* in each medium, refraction occurs. Consequently, the wave path from the element to the pixel is represented by two straight lines with distances *d_1_* and *d_2_* (or *d_3_* and *d_4_*), as shown in Figure 1. Thus, the time-of-flight can be written as:

(2) $$t_{e}=\frac{d_{1}}{c_{1}}+\frac{d_{2}}{c_{2}}\;;\;\;t_{r}=\frac{d_{4}}{c_{1}}+\frac{d_{3}}{c_{2}}$$

(3) $$t_{e}=\;\frac{\sqrt{{\left(x_{c}-X_{e}\right)}^{2}+{\left(s(x_{c})\right)}^{2}}}{c_{1}}+\frac{\sqrt{{\left(x-x_{c}\right)}^{2}+{\left(z-s(x_{c})\right)}^{2}}}{c_{2}}$$

(4) $$t_{r}=\;\frac{\sqrt{{\left(x_{c}-X_{r}\right)}^{2}+{\left(s(x_{c})\right)}^{2}}}{c_{1}}+\frac{\sqrt{{\left(x-x_{c}\right)}^{2}+{\left(z-s(x_{c})\right)}^{2}}}{c_{2}}$$

Nevertheless, to calculate the times-of-flight *t_e_* and *t_r_*, the correct paths should be selected based on the Fermat’s principle. In practice, this task consists of finding the transmission point (*x_c_*, *s*(*x_c_*)), on the surface *s*(*x*)*,* that yields a stationary time-of-flight:

(5) $$\frac{dt_{e}}{dx_{c}}=0;\;\frac{dt_{r}}{dx_{c}}=0$$

Moreover, the calculation of the times-of-flight for each pixel-element pair can be done only once since reciprocity is valid. That means that the time-of-flight from a pixel to an element is the same when it acts as an emitter or as a receiver. Throughout the paper, the surfaces were discretized using λ_water_/5 between samples, and a grid search method was used to find, between all surface points, the one that satisfies the condition in Equation (5).

To image each specimen, an internal region of interest was discretized into a grid of pixels. In each case, the grid of points was centered relative to the target center in the x-direction. Finally, the spacing between each point on the grid, or the pixel resolution, was set to 0.2 × 0.2 mm.

### 2.2. Probe Standoff Optimization Method

The reliability of the ultrasonic phased array total focusing method (TFM) imaging of parts with curved geometries also depends on the probe standoff. This is because some combinations of surface profile and standoff interfere in the phased array focusing capacity. Therefore, strong artifacts arise and resolution is lost in the imaging process, to the point that it is impossible to identify any defect indications with certainty. This paper, therefore, introduces a probe standoff optimization method (PSOM) to avoid these poor combinations. The main objective of the algorithm is to indicate the best probe standoff for a given set of curved surface geometry, defect location, probe and material parameters. Specifically, the algorithm improves the reliability of the imaging, ensuring the correct selection of standoff prior to the inspection. Moreover, it also may be used to predict the performance of the imaging. The imaging performance of an ultrasonic phased array may be predicted by the calculation of its point spread function (PSF). The PSF measures the array response to a point reflector [6]. This response is directly related to the phased array imaging performance. In an immersion inspection, the PSF is dependent on the array characteristics, media properties, interface shape and probe standoff. Therefore, the PSOM algorithm uses PSF characteristics to estimate the probe standoff that yields the optimal TFM image.

Many researchers have developed complete and accurate methods for the simulation of the array response, and hence the estimation of the PSF [6,25,26]. Based on these methods, a simplified PSF calculation was conducted in the present study. Since the aim of the PSOM is to find the best PSF of the TFM imaging using a single wave mode, a full simulation is not needed. Moreover, this work applied a PSF calculation that takes advantage of the outputs of the TFM described in Section 2.1. Thus, the pulse response of a point target *T*(*x*,*z*) inside the part is estimated by shifting and modifying a tone-burst, by using the probe parameters and the TFM outputs. This simplification results in a lower computational cost while maintaining a valid estimate of the system’s PSF.

First, the probe parameters, surface profile *s*(*x*), materials, point target *T*(*x*,*z*) and standoff must be defined. As shown in Figure 1, by applying the same TFM procedure described in Section 2.1, the paths from *e* to *T*(*x*,*z*) and to *r* are found. Then, the PSF estimation is based on the ray paths *d_1_*, *d_2_*, *d_3_*, *d_4_*, the angles *θ*_1_, *θ*_2_, *θ*_3_, *θ*_4_, and times-of-flight *t_e_* and *t_r_*. The times-of-flight *t_e_* and *t_r_* are used to shift a Dirac’s delta function. This delta function is then used to delay a tone-burst *P*[*t*] by a convolution operation:

(6) $$P\left[t\right]\;*\;\delta \left[t+t_{e}+t_{r}\right]=P\left[t+t_{e}+t_{r}\right].$$

Throughout the paper, *P*[*t*] is a Hann windowed tone-burst with 4 cycles and centered at 5 MHz. The convolution of *P*[*t*] yields the signal response estimate *u*(*e*,*r*,*t*) for this element combination:

(7) $$u\left(e,r,t\right)=P\left[t+t_{e}+t_{r}\right]$$

Now, it is necessary to account for the signal attenuation due to element directivity, the divergence of the ultrasonic wave and the transmission loss due to impedance mismatch:

(8) $$U\left(e,r,t\right)=u\left(e,r,t\right)\cdot G_{e}\cdot D_{e}\cdot T_{e}\cdot G_{r}\cdot D_{r}\cdot T_{r}$$

where *G_e_* is the geometric attenuation, *D_e_* is the element directivity and *T_e_* is the transmission coefficient in the emission and *G_r_* is the geometric attenuation, *D_r_* is the element directivity and *T_r_* is the transmission coefficient in the reception [23], defined as:

(9) $$G_{e}=\frac{1}{\sqrt{d_{1}+d_{2}}};\;G_{r}=\frac{1}{\sqrt{d_{3}+d_{4}}}$$

(10) $$D_{e}=\sin c\left(\frac{2v\pi f}{c_{1}}\cdot \sin\left(\theta_{1}\right)\right);\;D_{r}=\sin c\left(\frac{2v\pi f}{c_{1}}\cdot \sin\left(\theta_{4}\right)\right)$$

(11) $$T_{e}=\frac{2\rho_{2}c_{2}\cdot \cos\left(\theta_{1}\right)}{\rho_{1}c_{1}\cdot \cos\left(\theta_{2}\right)+\rho_{2}c_{2}\cdot \cos\left(\theta_{1}\right)};\;\;T_{r}=\frac{2\rho_{1}c_{1}\cdot \cos\left(\theta_{3}\right)}{\rho_{2}c_{2}\cdot \cos\left(\theta_{4}\right)+\rho_{1}c_{1}\cdot \cos\left(\theta_{3}\right)}$$

Here, *2v* is the length of the piezo element and *f* is the central frequency of the probe. In addition, *ρ*_1_ and *c*_1_ are, respectively, the density and the longitudinal wave speed of the first medium, *ρ*_2_ and *c*_2_ the density and the longitudinal wave speed of the second medium.

**Figure 2:**
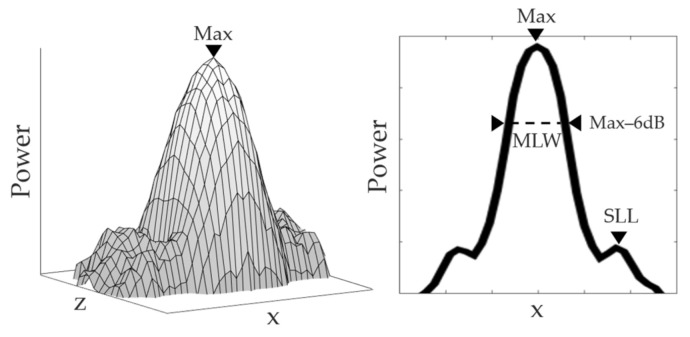
PSF example. The power represents the image intensity at a given point. *PSF*(*x*) is defined at *z*, where the power is maximum. Main lobe width and side lobe levels are found as illustrated.

With this calculation done for all possible combinations of emitter *e* and receiver *r*, the FMC due to the point target *T*(*x*,*z*) is estimated. Then, using the procedure described in Section 2.1, the times-of-flight of the pixels around the target are calculated. By using these values, *U*(*e*,*r*,*t*) is translated into a TFM image. The image intensity caused by a point reflector is the PSF of the imaging system:

(12) $$PSF\left(x,z\right)=\left|\underset{e}{\sum }\underset{r}{\sum }U(e,r,t_{e}+t_{r})\right|$$

which is exemplified by Figure 2. Here, the power represents the image intensity at a given (*x*,*z*) point. The maximum intensity is found at the exact point target position *T*(*x*,*z*). Moreover, since the lateral resolution is the scope of this analysis, the *PSF*(*x*,*z*) can be studied only at this *z* point, with it being reduced to *PSF*(*x*). In the *PSF*(*x*), as shown in Figure 2, the algorithm discriminates two important characteristics: the side lobe level (SLL) and the main lobe width (MLW). The SLL is found by a peak search method which looks for the highest value after the maximum in the *PSF*(*x*). On the other hand, the MLW is found by the −6 dB drop method. The MLW is the distance *x_q_–x_p_* between two points, where the *PSF*(*x*) is equal to the maximum subtracted by −6 dB:

(13) $$PSF\left(x_{q\;}\right)=PSF\left(x_{p\;}\right)=Max-6dB;\;MLW=x_{q\;}-x_{p\;}$$

The reasoning behind the choice of these two parameters lies in the image formation. The sharpness and precision of the TFM synthetic focus, which is ultimately the PSF, define the TFM image quality. Since the image is a convolution of the real target and the PSF, the MLW gives an estimate of the lateral resolution, while the SLL indicates the level of aberrations in the image [27]. Hence, the PSOM algorithm searches for the best tradeoff between the lateral resolution and the artifact level.

**Figure 3:**
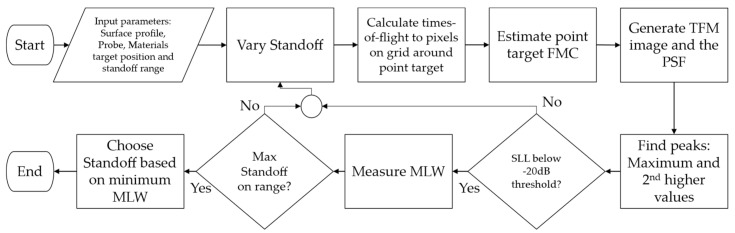
Flowchart of the Probe Standoff Optimization Method algorithm. A *PSF*(*x*) calculation and discrimination are conducted to find the standoff with the best tradeoff between the lateral resolution and the artifact level.

Now that the PSF calculation has been described, the flowchart of the algorithm is presented in Figure 3. The first step of the PSOM is to define all input parameters for the calculation. Then, having the standoff test range, the algorithm starts the PSF calculation at a given standoff, which is varied at each iteration. With the TFM procedure, the times-of-flight and path parameters are calculated. In the sequence, the FMC is estimated based on these values and then translated into the image of the *PSF*(*x*,*z*). The peaks are found and the SLL is calculated. The SLL is measured and verified to see if it is below the defined threshold, (−20 dB here), which was verified as an acceptable image quality. If the SLL is above the threshold, the standoff is varied. Otherwise, the MLW is measured. As such, the algorithm does not waste time computing the MLW for poor image candidates. After the last standoff is used in calculation, the algorithm chooses the standoff with the lower MLW as the optimal probe standoff.

The algorithm was run for all experimental parameters described in the next section.

### 2.3. Experimental Analysis

**Figure 4:**
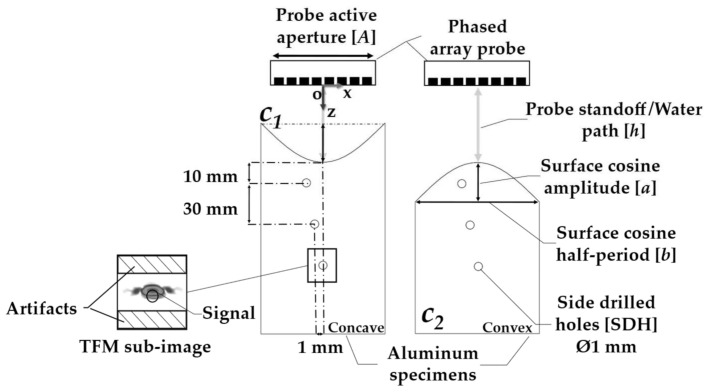
Schematic of the sinusoidal concave and convex specimens inspected by immersion phased array ultrasonic testing. The probe is centered relative to the surface. Three SDH are positioned inside the specimens and are analyzed by the TFM sub-image shown.

Experiments were carried out to verify the validity of the PSOM. First, a set of sinusoidal surface profiles was defined for the machined aluminum specimens. The sinusoidal shape was chosen to create a challenging variable wave incidence scenario, while maintaining a generic approach. Figure 4 presents a schematic of the specimens with sinusoidal top surfaces under immersion inspection. Here, the specimens were divided and named according to their surface curvature. The surfaces of the concave ones are described by:

(14) s∪x=h−a+a·cosπxb; {x|−b2≤x≤b2}

additionally, the convex surfaces were written as:

(15) s∩x=h+a−a·cosπxb; {x|−b2≤x≤b2}

where *h* is the probe standoff or water path, *a* is the surface cosine amplitude and *b* is the half-period of the cosine function, as shown in Figure 4.

The specimen parameters, cosine amplitude *a*, half-period *b*, thickness *l* and probe standoff range *h*, are presented in Table 1. A flat surface was manufactured for the benchmark imaging, with two pairs of concave and convex surfaces to represent a weak and a strong curvature sharpness relative to the probe aperture. A set of cosine amplitudes *a* was chosen for this study, varying from zero λ_Al_, representing a flat surface, to 15 λ_Al_. Different curvatures enabled the analysis of how combinations of surface curvature and probe standoffs influence the inspection. The materials selected were water, for the immersion medium, and aluminum, for the specimen. The longitudinal wave velocity of the water *c*_1_ and of the aluminum *c*_2_ are written in Table 2, along with the material’s respective densities *ρ*_1_ and *ρ*_2_. Here, λ_Al_ is the wavelength in the specimen material at the center frequency chosen for the inspection (5 MHz).

With the surface profiles defined, the variation range of the probe standoff *h* was chosen. As shown in Table 1, *h* was varied, ranging between 20 and 130 mm, with a 10 mm step. Both the phased array probe and the specimen were centered relative to the origin O, as shown in Figure 4. It can be noted that *h* is defined as the distance in *z* from the center point of the transducer to the center of the surface profile. This ensured approximately the same propagation distance from the probe to the subsurface flaws for both concave and convex specimens.

In this study, the side-drilled holes were positioned within the specimens according to the schematics presented in Figure 4. Three side-drilled holes (SDH) with a diameter of 1 mm were selected as the inspection targets. The size of the flaws was designed to be smaller than λ_Al_ at 5 MHz, but still above the theoretical sizing limit of a half-wavelength. In addition, different defect depths, ranging from 10 to 70 mm, were designed to evaluate the effect of the surface shape on penetration loss. Finally, the defects were laterally offset by 1 mm between one another to avoid shadowing.

**Figure 5:**
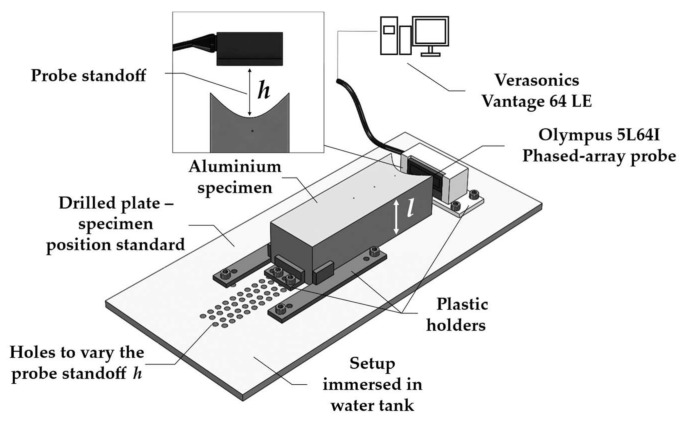
Schematic of the experimental setup used to validate the optimization calculations. The setup was assembled using 3D-printed plastic holders.

As can be seen in Figure 5, the experimental setup comprised a Verasonics Vantage 64 LE array controller and a 64-element 5 MHz Olympus 5L64I phased array probe. The parameters of the latter are shown in Table 2. For each specimen, the setup was assembled using 3D-printed plastic holders and an aluminum plate with holes. This setup worked as a standard to fasten both the specimen and the phased array transducer in the correct centered positions and *h* range.

With the assembled setup immersed in a water tank, FMC were acquired for all probe standoffs *h*. The standoff was increased by changing the position of the specimen’s holder to a lower hole on the aluminum plate, as shown in Figure 5. A 25-volt four-cycle Hann windowed burst centered at 5 MHz was used as the input signal. Acquisition was done at a sampling frequency of 62.5 MHz and 30 averages were performed. In addition, a bandpass filter with cut-off frequencies of 3 and 7 MHz was applied to the resulting time traces. All the FMC data was extracted and post-processed on an TFM algorithm written in Matlab^®^.

#### Data Post-Processing and Metrics

The FMC data was post-processed using the total focusing method (TFM) procedure described in Section 2.1. In this case, the image reconstruction using the TFM accounted for the known surface profiles and did not require their prior identification. This assumption was made because this work aims to investigate the isolated effect of the surface profile and the probe standoff on imaging.

A quantitative analysis of the TFM images was then carried out, with a separate analysis conducted for each SDH, as in in Figure 4. Two metrics were selected to evaluate the images: the signal-to-artifact ratio (SAR) and the array performance indicator (API) [19].

The TFM images were generated using the longitudinal mode and accounting only for direct paths. Hence, the reflection coming from the top face of the side-drilled holes is the stronger indication expected in the images.

However, due to the curvatures on the surfaces, a loss of coherence and focusing capacity may occur, thus creating image aberrations and making it hard to identify defects. For this reason, the SAR was introduced as a metric to measure the level of these image artifacts, accounting for changes in the reflector amplitude relative to the level of aberrations. For each reflector, the sub-image area was divided into two regions, one containing the signal and the other, the possible surrounding artifacts, as shown in Figure 4.

This region was selected large enough to contain the echoes from the longitudinal waves reflecting on the top or internal surfaces of the SDH and possible creep waves. The maximum amplitude value *I_max_* in the signal region represents the signal on the SAR calculation in Equation (16). Consequently, the remaining pixels in the sub-image area represent the control region for the level of artifacts, as shown by the hatched region in Figure 4. These pixels have an intensity *I_artifacts_*. In the experiments, random and structural noises are present, which reduces the SAR. The SAR was calculated by taking the *I_max_* and dividing it by the root mean square of *I_artifacts_*. This ratio was put on a decibel scale to yield the SAR:

(16) $${SAR=20\;\log}_{10}\left(\frac{I_{max}}{\sqrt{{<I}_{\mathrm{artifacts}}^{2}>}}\right)$$

On the other hand, the API was chosen to evaluate how the lateral resolving capacity of the imaging system would be affected by the combination of inspection parameters. The pixels (*I_−_*_6*dB*_), with intensities equal to the maximum and up to 6 dB below the maximum intensity (*I_max_* − 6 dB ≤ *I_−_*_6*dB*_ ≤ *I_max_*), were considered as the area *A_−6dB_* of the reflector in the image. Hence, the number of pixels within this range multiplied by the image resolution of 0.2 × 0.2 mm yielded the area size *A*_−*6dB*_. The API was calculated for each sub-image using Equation (17). For each case, the ratio between the area (*A_−6dB_*) and the square of the wavelength (λ^2^_Aluminum_) was calculated:

(17) $$API=\frac{A_{-6\mathrm{dB}}}{\lambda_{\mathrm{Al}}^{2}}$$

## 3. PSOM and Experimental Results

**Figure 6:**
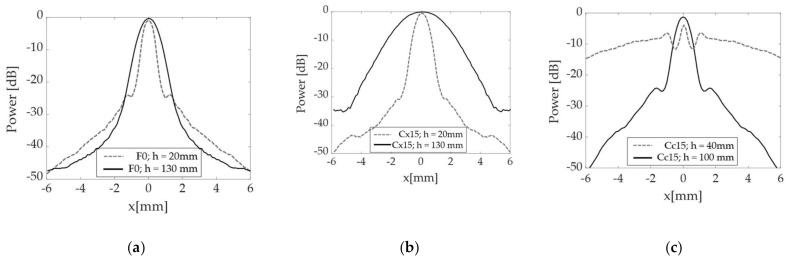
PSF(x) calculated for a target positioned 10 mm deep inside the parts: (**a**) Flat specimen F0 at two probe standoffs *h* = 20 and 130 mm; (**b**) Convex specimen Cx15 at two probe standoffs *h* = 20 and 130 mm; (**c**) Concave specimen Cc15 at two probe standoffs *h* = 40 and 100 mm.

First, some numerical predictions are presented through the PSFs generated using the PSOM. Figure 6 contain the PSF comparison between different surfaces and standoffs for the SDH positioned 10 mm deep into the parts. It is shown how the PSF behaves in the two extremes of the range, *h* = 20 and 130 mm, for the F0 specimen in Figure 6a and for the Cx15 specimen in Figure 6b. Additionally, in Figure 6c, the PSF is shown for the Cc15 specimen at *h* = 40 and at 100 mm standoffs to illustrate a poor and a high imaging performance.

Little variation is observed in Figure 6a for the F0 specimen, with a slight increase in MLW and reduction in SLL. However, since the SLL is below −20 dB, no imaging artifacts will appear in the imaging. On the other hand, the Cx15 specimen, Figure 6b, presents a large MLW increase over the same range. While the performance is comparable to the F0 benchmark at *h* = 20 mm, the loss of lateral resolution is latent at the 130 mm standoff. Finally, the Cc15 in Figure 6c presents a different behavior. At the 40 mm standoff, the SLL is as high as −9 dB, which indicates a very poor focus. Consequently, it is expected that the image of a reflector would be greatly compromised by artifacts. Meanwhile, at *h* = 100 mm, the SLL drops to −22 dB, indicating a much better focus definition.

**Figure 7:**
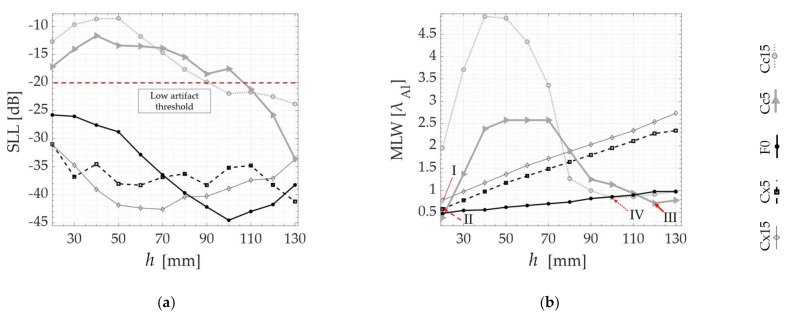

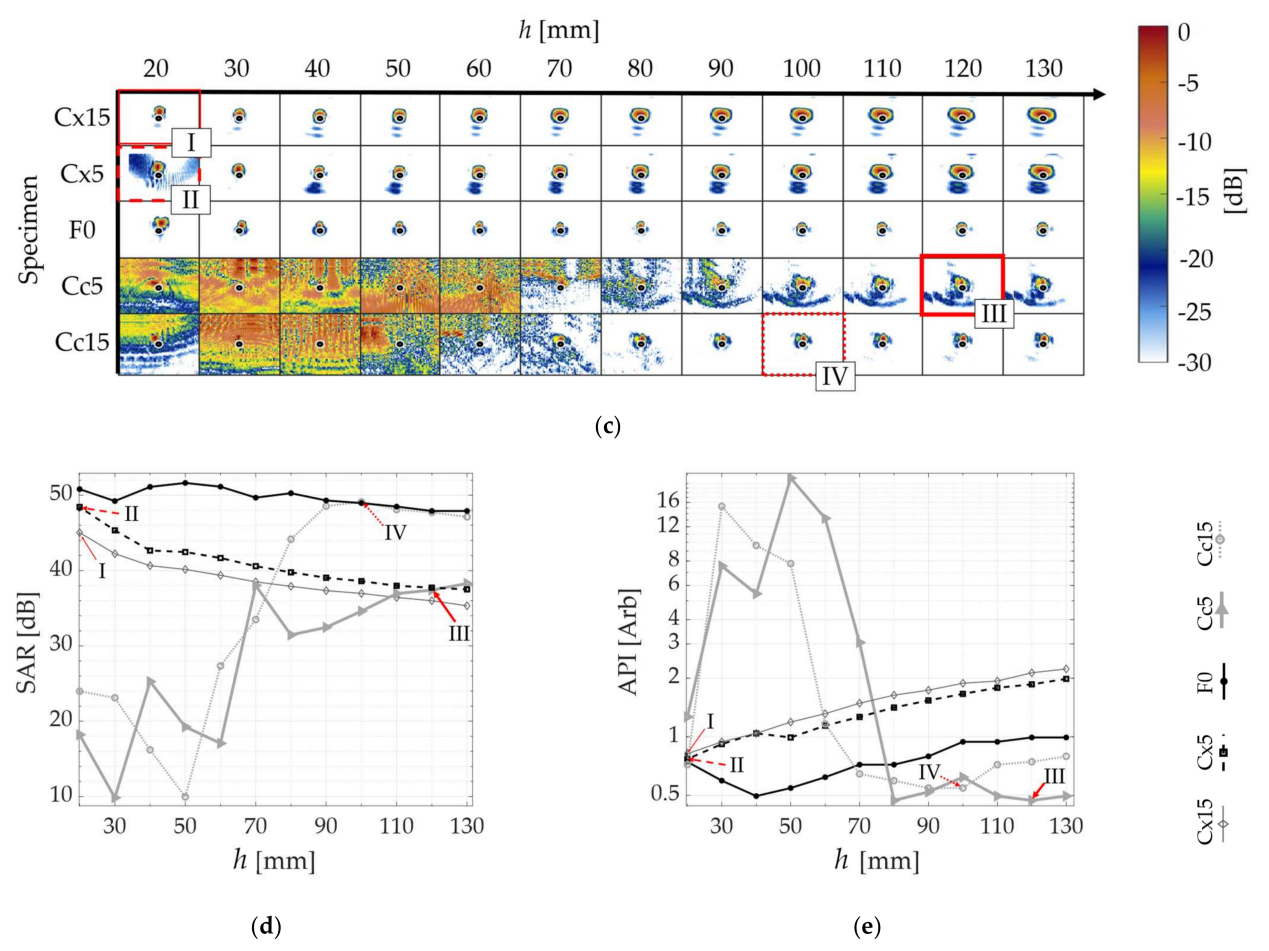
PSOM and experimental results from the SDH at 10 mm depth inside each specimen: (**a**) SLL obtained from *PSF*(*x*) at each standoff *h*; (**b**) MLW vs. *h*; (**c**) TFM images at different probe standoffs *h* in each of the five specimens; (**d**) SAR vs. standoff; (**e**) API vs. standoff. The numbers I, II, III and IV indicate the optimal probe standoffs selected by the PSOM.

**Figure 8:**
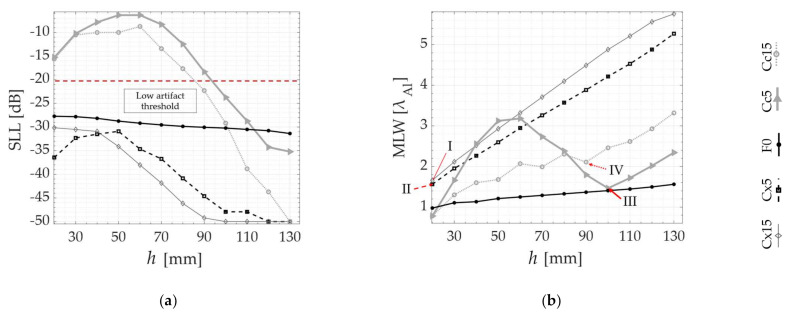

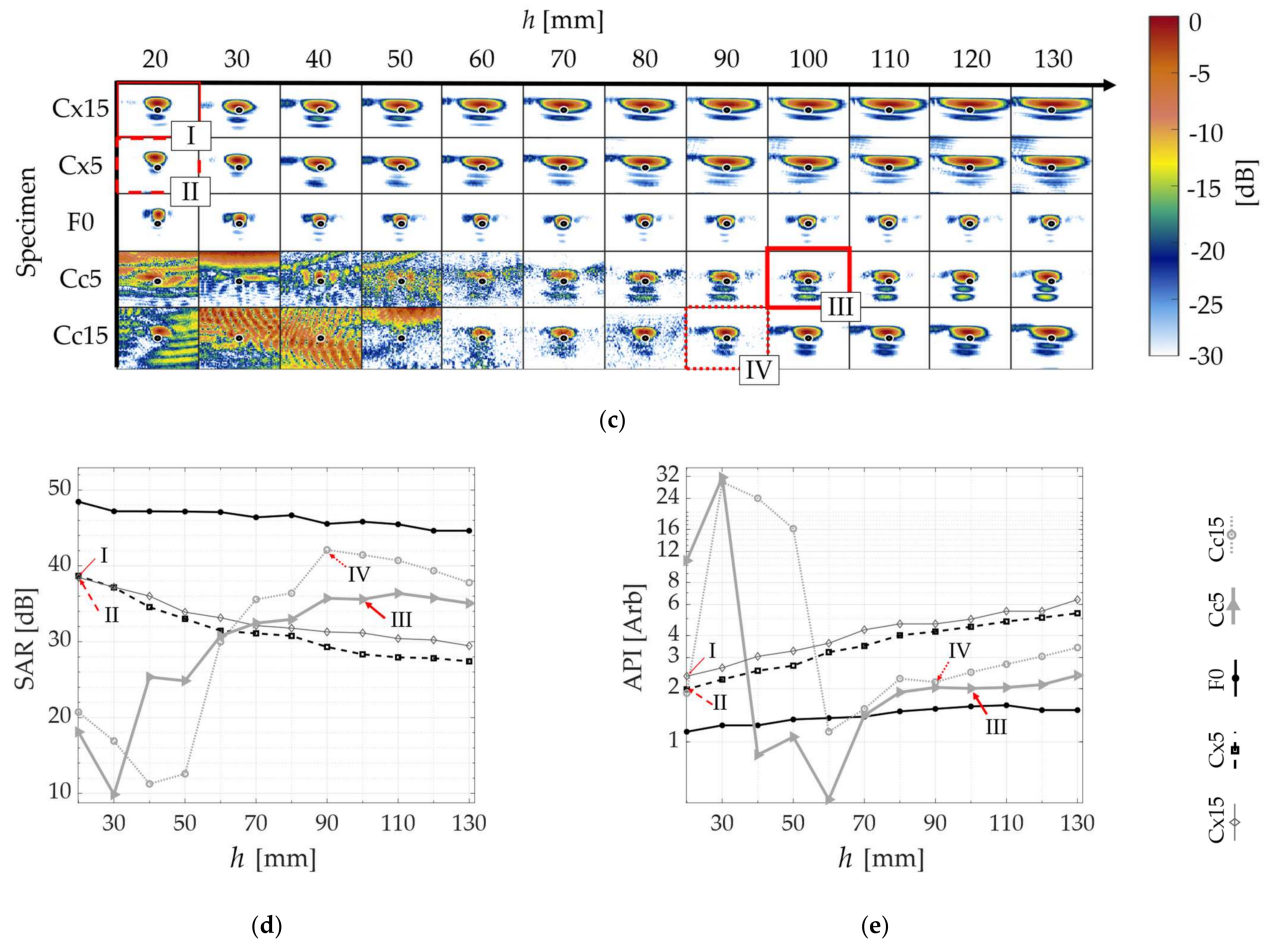
PSOM and experimental results from the SDH at 40 mm depth inside each specimen: (**a**) SLL obtained from *PSF*(*x*) at each standoff *h*; (**b**) MLW vs. *h*; (**c**) TFM images at different probe standoffs *h* in each of the five specimens; (**d**) SAR vs. standoff; (**e**) API vs. standoff. The numbers I, II, III and IV indicate the optimal probe standoffs selected by the PSOM.

**Figure 9:**
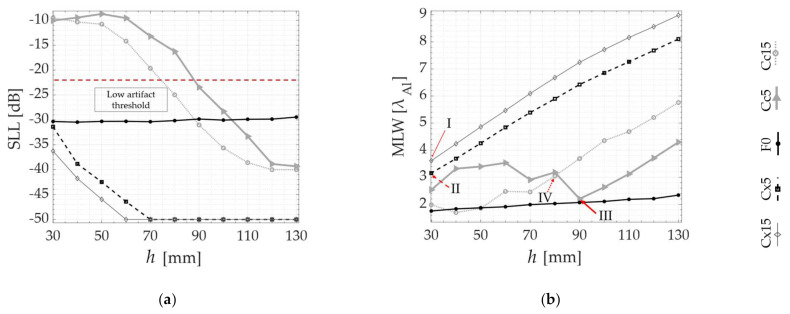

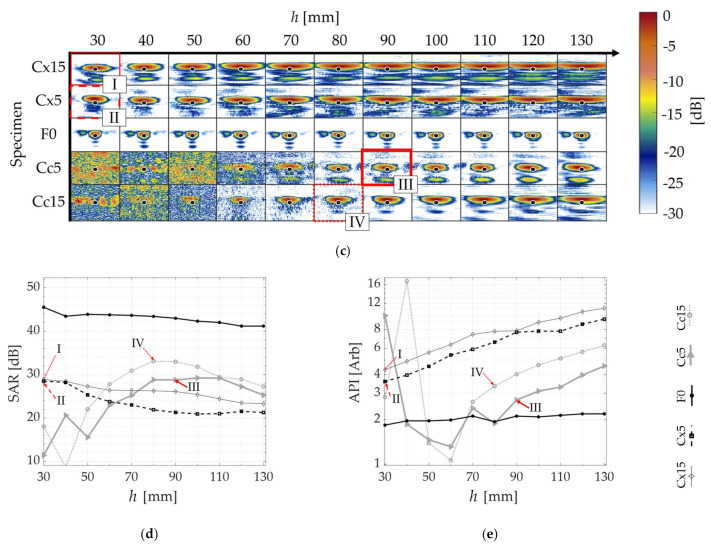
PSOM and experimental results from the SDH at 70 mm depth inside each specimen: (**a**) SLL obtained from *PSF*(*x*) at each standoff *h*; (**b**) MLW vs. *h*; (**c**) TFM images at different probe standoffs *h* in each of the five specimens; (**d**) SAR vs. standoff; (**e**) API vs. standoff. The numbers I, II, III and IV indicate the optimal probe standoffs selected by the PSOM.

In the following, the SLL and MLW are plotted as a function of all probe standoffs *h*, respectively, in Figure 7a,b, Figure 8a,b and Figure 9a,b. Although the algorithm does not compute the MLW below the −20 dB threshold, it is still presented as a way of verifying the agreement between the PSF and the experimental TFM images. Figure 7 contains the results for the SDH positioned at 10 mm inside the part, while Figure 8 and Figure 9 contain the results for the 40 and 70 mm deep SDHs. A dotted line is used to show the SLL threshold at −20 dB in all SLL plotting. In all the graphs, the results are overlayed for all specimens tested as in Table 1, as follows: Concave (Cc5 and Cc15), Convex (Cx5 and Cx15) and Flat (F0). Finally, the numbers I (Cx15), II (Cx5), III (Cc5) and IV (Cc15) are used to indicate the values of SLL and MLW of the optimal probe standoff selected by the PSOM. The PSOM algorithm run time on Matlab^®^ 2021a for each combination of flaw position and surface profile was approximately 400 s, considering 12 different probe standoffs. The setup used for these computations had 32 Gb of RAM and an Intel^®^ Core™ i7-8086k CPU @4.00GHz.

To verify these predictions, the results of the validation experiments are also presented in Figure 7c, Figure 8c and Figure 9c, where the TFM images of the side-drilled holes are composed into matrices. In these TFM matrices, the top surface shape, referenced by the specimen number in Table 1, is identified in the vertical axis and the probe standoff *h* varies along the horizontal axis. In addition, the TFM images of a specimen with a flat top surface are plotted on the middle line, to serve as the image quality standard. The black circles in the middle of each image represent the real side-drilled hole position and size. Again, each matrix, from Figure 7, Figure 8 and Figure 9 refers to each defect depth studied. Consistently, the same numbers I, II, III and IV refer to the optimal probe standoff in the TFM image grid. It is important to mention that the probe standoff *h* = 20 mm was suppressed for the defects at 70 mm, due to the interference of the front-wall reflections.

The quality of the qualitative TFM images shown is described by the graphs presented in Figure 7d,e, Figure 8d,e and Figure 9d,e. The SAR and the API are presented as a function of the probe standoff *h* for the Concave (Cc5 and Cc15), Convex (Cx5 and Cx15) and Flat (F0) top surfaces studied. The SAR is plotted in Figure 7d, Figure 8d and Figure 9d. Similarly, Figure 7e, Figure 8e and Figure 9e show the API. Again, the optimal probe standoff values of SAR and API are indicated by the numbers I, II, III and IV.

A good agreement is observed as the behavior predicted by the *PSF(x)* estimation is repeated in the experiments. In each case, from Figure 7, Figure 8 and Figure 9, the TFM images of the flat benchmark F0, and their SARs and APIs, are practically constant for most standoffs *h* analyzed. Meanwhile, the convex and concave cases each present a different behavior as a function of the change of the probe position relative to the surface.

Analyzing the images from defects under the Cx5 and Cx15 surfaces in Figure 7c, Figure 8c and Figure 9c, the area of pixels with intensities between 0 and −6 dB (A_−6dB_) gets larger as the standoff *h* increases. Quantitatively, the graphs in Figure 7d,e, Figure 8d,e and Figure 9d,e demonstrate a consistent SAR drop and an API increase as a function of an increase in the standoff *h*, for all defect depths, in the case of convex top surfaces. This confirms the *PSF*(*x*) trends of MLW increasing with *h*. Therefore, the PSOM indicates the minimum standoff in all convex cases, where SLL is below the threshold and the MLW is minimized. At the standoffs I and II, the SAR is maximum, and the API is minimum.

Furthermore, the images generated below the Cc5 and Cc15 profiles, presented in Figure 7c, Figure 8c and Figure 9c, show a different behavior. The TFM images are full of reconstruction artifacts at certain probe standoffs *h*. At these probe positions, it becomes impossible to identify the presence of the reflector inside the specimen. The SLL verified from Figure 7a, Figure 8b and Figure 9a agree with the TFM results, being above the threshold for all these standoffs. Hence, the PSOM discarded these standoffs, and based on the minimum MLW, selected the optimal standoffs III and IV in all defect depths. At the optimal points III and IV, the SAR and the API have values consistent with the tradeoff between the resolution and artifact level. In some cases, the maximum SAR occurs for an also high API, and, therefore, a lower resolution is chosen while keeping the artifact level in the image low.

**Figure 10:**
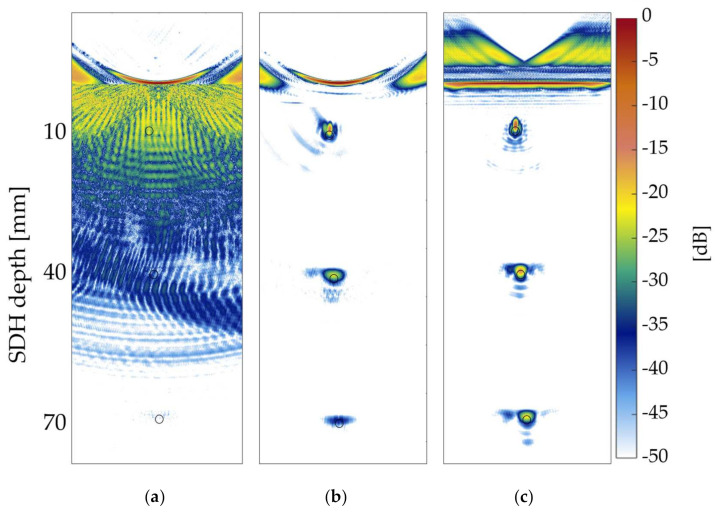
TFM image of the three side drilled holes and the specimens’ surfaces: (**a**) Concave specimen Cc15 at the probe standoff *h* = 40 mm; (**b**) Concave specimen Cc15 with the PSOM applied to each flaw; (**c**) Benchmark flat specimen F0 at the probe standoff *h* = 40 mm.

Finally, the images presented in Figure 10a–c present a comparison to verify the overall performance of the PSOM for the three side drilled holes. In these TFM images, the surface profile reconstructed through the imaging process is shown for reference. The amplitude of all three images was normalized by the front wall amplitude in Figure 10c, which contains the image of the flat specimen benchmark F0. In all Figure 10a–c, the black circles represent the real size and positions of the SDHs. Figure 10a contains the TFM image of the concave specimen Cc15 at the probe standoff *h* = 40 mm. Additionally, in Figure 10a, strong artifacts are present blurring the image, which makes it impossible to identify any indications of the internal flaws. However, in Figure 10b, the TFM of the same specimen Cc15 was reconstructed using the PSOM for each flaw. When using the PSOM, all three flaws have their indications resolved in the image, similarly to what is observed in the flat benchmark in Figure 10c. This illustrates the relevance of the algorithm and the design of the optimal probe standoff. The flaw resolving capacity of the TFM applied to curved specimens is directly related to the optimization process proposed.

## 4. Discussion

The explanation behind the results obtained resides in the nature of the TFM. This imaging method is a synthetic focusing procedure that beamforms the FMC signal into fully focused images. This means the phased array is delayed to focus on every point in a region of interest. Thus, the image quality depends on the physical capacity of the array to generate a focus inside the specimen. In an immersion setup, this focus will be influenced by the curvature of the interface and its distance from the probe. In the case of curved surfaces, the interface will act as a lens, changing the focus shape. Moreover, even though the algorithm forces the focusing to occur on a given point, physically, the directivity of the probe elements limits the capacity of the array to generate a sharp focus. Ultimately, to focus on a given point, the waves will sometimes travel through paths with angles of refraction that are in directions of low emission/reception power on the probe. That is exactly what the PSOM ultimately accounts for: the effect of varying the wave paths and angles of refraction on the focus capacity. The imaging quality will be determined by the SLL levels and the resolution by the MLW. The optimization algorithm selects the standoff that generates a focus with a low SLL and minimizes the MLW, and these two parameters are directly related to the image quality metrics, API and SAR. The SLL indicates the level of lateral lobes in the focus that cause artifacts, and finally, the level of SAR, while the MLW indicates the resolution of the focus for a single point, and, therefore, is directly related to the API in the TFM images.

The latter is confirmed by the results, which show that the imaging performance strongly depends on the top surface curvature, the defect position, and most importantly, the probe standoff. In each case, from Figure 7, Figure 8 and Figure 9, the TFM images of the flat benchmark, as well as their SARs and APIs, present negligible variations when compared to the images of curved parts.

The present study demonstrated that the TFM imaging of defects inside concave parts Cc5 and Cc15 achieves a better performance at an optimal probe standoff *h*. This can be found prior to the inspection using the presented PSOM. In the case of Figure 7, at the optimal *h* indicated by IV, the image SAR gained 33 dB when compared to a poor standoff. If the PSOM is not applied, the strong image artifacts that occur at some standoffs and concave surface curvature combinations will make it impossible to identify defects. Additionally, the best tradeoff between the resolution and image quality was obtained using the proposed method. This means that the optimal standoff presented the maximum SAR at the cost of a slightly increased API. After the optimal standoff, the API has an ascending tendency while the SAR values drop. It is important to note that depending on the application, the standoff selection criteria may be modified, favoring lateral resolution for cases with close defects or favoring SAR for highly attenuating materials.

Additionally, from the results, it is verified that the TFM images of convex Cx5 and Cx15 specimens lose performance as the standoff is increased. This agrees with the *PSF* prediction, where the MLW demonstrates how the focal point loses lateral resolution with an increase in the standoff. Hence, the PSOM indicates the standoff with the minimum MLW for all convex cases, since the SLL was lower than the threshold. For example, in Figure 9, the optimal standoff I presents an API slightly above 4, which is higher than the benchmark of 2, showing the influence of the curvature on the loss of resolution. Still, if a standoff of 130 mm is used, the Cx15 will have a drop in resolution of about 11 API. Finally, Figure 10a–c illustrated the relevance of the algorithm and the importance of designing the optimal probe standoff. Without the PSOM, the flaw identification reliability of the TFM on curved specimens becomes compromised.

## 5. Conclusions

This study demonstrated how the performance of the phased array immersion inspection could be optimized based on the probe standoff, using an optimization method (PSOM). The PSOM capacity to calculate the best probe standoff was verified through the good agreement between its predictions and the results of the experimental phased array imaging of parts with concave and convex top surfaces. The results confirmed that the optimization process greatly improves the imaging performance of curved parts.

The TFM imaging of defects inside concave and convex sinusoidal parts achieved a better performance at an optimal probe standoff *h*. In the case of concave parts, the PSOM ensured the selection of an optimal standoff, which avoids the appearance of strong artifacts while keeping a good lateral resolution. In the case of convex parts, a simpler scenario was found by the PSOM, where the best standoff was the minimum without front-wall second reflections.

Nevertheless, it must be noted that the ultrasound penetration and performance suffer due to the surface profile, and that the inspection depth and image quality are reduced accordingly. Future work must focus on the calibration issues caused by the curved surface because of the variability of the imaging performance. For example, the algorithm may be used as a fast tool to predict the resolution based on the surface profile. In addition, this work analyzed convex and concave surfaces separately. However, in real applications, it is common to find complex surfaces formed of combinations of concave and convex curvatures. Therefore, future works should investigate the validity of the PSOM algorithm for such surfaces.

## Figures and Tables

**Table 1:** Specimen surface curvature parameters and probe standoffs.

Specimen	Surface	*a* (λ_Al_)	*b* (mm)	*l* (mm)	*h* (mm)
Cx15	Convex $s_{\cap }\left(x\right)$	15	60		
Cx5	Convex $s_{\cap }\left(x\right)$	5	38.4		
F0	Flat $s_{-}\left(x\right)$	0	62	60	{20, 30, …, 130}
Cc5	Concave $s_{\cup }\left(x\right)$	5	38.4		
Cc15	Concave $s_{\cup }\left(x\right)$	15	60		

**Table 2:** Probe and material parameters.

**Aperture *A* (mm)**	**Frequency *f* (MHz)**	**Pitch (mm)**	**2*v* (mm)**
38.4	5	0.6	0.55
**λ_Al_ (mm)**	**Elevation (mm)**	**Bandwidth**	**Element Count**
1.27	10	83%	64
***c*_1_ (m/s)**	***ρ*_1_ (g/cm^3^)**	***c*_2_ (m/s)**	***ρ*_2_ (g/cm^3^)**
1480	1	6470	2.77

## Data Availability

Data and codes used in this study are openly available at https://pulets.ca/open-data/sensors-21-06665.

## References

[1] Mahaut S., Roy O., Beroni C., Rotter B. (2002). Development of phased array techniques to improve characterization of defect located in a component of complex geometry. Ultrasonics.

[2] Hunter A.J., Drinkwater B.W., Wilcox P.D. (2010). Autofocusing ultrasonic imagery for non-destructive testing and evaluation of specimens with complicated geometries. NDT E Int..

[3] Sutcliffe M., Weston M., Charlton P., Donne K., Wright B., Cooper I. (2013). Full matrix capture with time-efficient auto-focusing of unknown geometry through dual-layered media. Insight-Non-Destr. Test. Cond. Monit..

[4] Honarvar F., Varvani-Farahani A. (2020). A review of ultrasonic testing applications in additive manufacturing: Defect evaluation, material characterization, and process control. Ultrasonics.

[5] Brown R.H., Dobson J., Pierce S.G., Dutton B., Collison I. (2017). Quantifying performance of ultrasonic immersion inspection using phased arrays for curvilinear disc forgings. AIP Conference Proceedings.

[6] Drinkwater B.W., Wilcox P.D. (2006). Ultrasonic arrays for non-destructive evaluation: A review. NDT E Int.

[7] Lane C.J. (2014). The inspection of curved components using flexible ultrasonic arrays and shape sensing fibres. Case Stud. Nondestruct. Test. Eval..

[8] Chatillon S., Cattiaux G., Serre M., Roy O. (2000). Ultrasonic non-destructive testing of pieces of complex geometry with a flexible phased array transducer. Ultrasonics.

[9] Russell J., Long R., Cawley P. (2010). Development of a membrane coupled conformable phased array inspection capability. AIP Conference Proceedings.

[10] Simonetti F., Fox M. (2019). Experimental methods for ultrasonic testing of complex-shaped parts encased in ice. NDT E Int..

[11] Sutcliffe M., Weston M., Dutton B., Cooper I., Donne K. Real-time full matrix capture with auto-focusing of known geometry through dual layered media. Proceedings of the NDT 2012 Conference in British Institute of Nondestructive Testing.

[12] Zhang J., Drinkwater B.W., Wilcox P.D. (2014). Efficient immersion imaging of components with nonplanar surfaces. IEEE Trans. Ultrason. Ferroelectr. Freq. Control.

[13] Kerr W., Pierce S.G., Rowe P. (2016). Investigation of synthetic aperture methods in ultrasound surface imaging using elementary surface types. Ultrasonics.

[14] Matuda M.Y., Buiochi F., Adamowski J.C. (2019). Experimental analysis of surface detection methods for two-medium imaging with a linear ultrasonic array. Ultrasonics.

[15] Le Jeune L., Robert S., Prada C. (2016). Plane wave imaging for ultrasonic inspection of irregular structures with high frame rates. AIP Conference Proceedings.

[16] Rachev R.K., Wilcox P.D., Velichko A., McAughey K.L. (2020). Plane Wave Imaging Techniques for Immersion Testing of Components With Nonplanar Surfaces. IEEE Trans. Ultrason. Ferroelectr. Freq. Control.

[17] Cruza J.F., Camacho J., Mateos R., Fritsch C. (2019). A new beamforming method and hardware architecture for real time two way dynamic depth focusing. Ultrasonics.

[18] Hoyle E., Sutcliffe M., Charlton P., Rees J. (2018). Virtual source aperture imaging with auto-focusing of unknown complex geometry through dual layered media. NDT E Int..

[19] Holmes C., Drinkwater B.W., Wilcox P.D. (2005). Post-processing of the full matrix of ultrasonic transmit–receive array data for non-destructive evaluation. NDT E Int..

[20] Camacho J., Atehortua D., Cruza J.F., Brizuela J., Ealo J. (2018). Ultrasonic crack evaluation by phase coherence processing and TFM and its application to online monitoring in fatigue tests. NDT E Int..

[21] Malkin R.E., Franklin A.C., Bevan R.L.T., Kikura H., Drinkwater B.W. (2018). Surface reconstruction accuracy using ultrasonic arrays: Application to non-destructive testing. NDT E Int..

[22] McKee J.G., Wilcox P.D., Malkin R.E. (2019). Effect of surface compensation for imaging through doubly-curved surfaces using a 2D phased array. AIP Conference Proceedings.

[23] Schmerr L. (2015). Fundamentals of Ultrasonic Phased Arrays.

[24] Long R., Russell J., Cawley P. (2012). Ultrasonic phased array inspection using full matrix capture. Insight-Non-Destr. Test. Cond. Monit..

[25] Chiao R.Y., Thomas L.J. (1994). Analytic evaluation of sampled aperture ultrasonic imaging techniques for NDE. IEEE Trans. Ultrason. Ferroelectr. Freq. Control.

[26] Lingvall F., Olofsson T., Stepinski T. (2003). Synthetic aperture imaging using sources with finite aperture: Deconvolution of the spatial impulse response. J. Acoust. Soc. Am..

[27] Ilovitsh A., Ilovitsh T., Ferrara K.W. (2019). Multiplexed ultrasound beam summation for side lobe reduction. Sci. Rep..

